# Using Knock-Out Mutants to Investigate the Adhesion of *Staphylococcus aureus* to Abiotic Surfaces

**DOI:** 10.3390/ijms222111952

**Published:** 2021-11-04

**Authors:** Christian Spengler, Friederike Nolle, Nicolas Thewes, Ben Wieland, Philipp Jung, Markus Bischoff, Karin Jacobs

**Affiliations:** 1 Experimental Physics and Center for Biophysics, Saarland University, 66123 Saarbrücken, Germany; c.spengler@physik.uni-saarland.de (C.S.); f.nolle@physik.uni-saarland.de (F.N.); nicolas.thewes@web.de (N.T.); 2 Institute of Medical Microbiology and Hygiene and Center for Biophysics, Saarland University, 66421 Homburg, Germany; ben.wieland@uks.eu (B.W.); philipp.jung@uks.eu (P.J.); markus.bischoff@uks.eu (M.B.); 3 Max Planck School Matter to Life, Jahnstraße 29, 69120 Heidelberg, Germany

**Keywords:** bacterial adhesion, single-cell force spectroscopy, *Staphylococcus aureus*, *Staphylococcus aureus* knock-out mutants, surface charge, hydrophobicity

## Abstract

The adhesion of *Staphylococcus aureus* to abiotic surfaces is crucial for establishing device-related infections. With a high number of single-cell force spectroscopy measurements with genetically modified *S. aureus* cells, this study provides insights into the adhesion process of the pathogen to abiotic surfaces of different wettability. Our results show that *S. aureus* utilizes different cell wall molecules and interaction mechanisms when binding to hydrophobic and hydrophilic surfaces. We found that covalently bound cell wall proteins strongly interact with hydrophobic substrates, while their contribution to the overall adhesion force is smaller on hydrophilic substrates. Teichoic acids promote adhesion to hydrophobic surfaces as well as to hydrophilic surfaces. This, however, is to a lesser extent. An interplay of electrostatic effects of charges and protein composition on bacterial surfaces is predominant on hydrophilic surfaces, while it is overshadowed on hydrophobic surfaces by the influence of the high number of binding proteins. Our results can help to design new models of bacterial adhesion and may be used to interpret the adhesion of other microorganisms with similar surface properties.

## 1. Introduction

*Staphylococcus aureus* is an opportunistic pathogen associated with different community and hospital acquired infections [1]. One reason for its high infectivity is that the cells can attach to various surfaces to form multicellular aggregates embedded in an extracellular matrix, called biofilms, in which the cells are protected against many therapeutics and the human immune system [2,3,4,5,6]. Therefore, the adhesion of cells and biofilm formation can take place on biotic surfaces as well as on abiotic surfaces, such as implanted medical devices. Due to the latter, the organism is a major cause of implant-related infections with severe consequences for the patient’s health [7,8,9,10,11,12]. Hence, understanding and controlling the adhesive behavior of *S. aureus* is of fundamental importance for health care and engineering [13,14].

The state-of-the-art method in quantitative bacterial adhesion research is AFM-based force spectroscopy with single bacterial probes (‘single-cell force spectroscopy’, SCFS) [15,16,17,18,19,20]. This method allows for the investigation of many different mechanisms on a single-cell or even molecular level. For instance, it can be performed on bare or conditioned surfaces, biotic or abiotic, as well as with pretreated cells [21,22,23,24,25].

As demonstrated by SCFS, bacterial adhesion to hydrophobic surfaces is governed by cell wall macromolecules that tether to the surface [6,26,27,28]. As a consequence, the adhesive strength of a single cell is determined by the number of contact-forming macromolecules and by the strength of each individual binding site, whereby one macromolecule could also have more than one of these binding sites. In other words, the adhesive energy is the integral of the negative part of a force–distance curve upon retraction. Of note, important adhesion parameters, such as the bacterial contact area to solid surfaces, highly depend on the individual cell [29] as well as on the position at the cell wall [30]. Thus, bacterial adhesion and its strength is—to a large extent—cell-individual and, therefore, general statements concerning the adhesion of certain cell types can only be derived with good statistics.

Macromolecules play a crucial role in adhesion to the natural environment and are important adhesion factors [31,32,33]. For instance, it was found that *S. aureus* cells lacking cell wall teichoic acids adhere more weakly to human endothelial [31] and epithelial cells [32] than wild-type bacteria and that surface anchored proteins are important for nasal colonization [33]. Other work demonstrated that individual adhesion factors such as fibronectin-binding protein A and clumping factor B are important for the ability of *S. aureus* to adhere to endothelial cells and to human-desquamated nasal epithelial cells, respectively [34,35].

Nevertheless, apart from the studies showing reduced adhesion of *S. aureus* cells lacking cell wall teichoic acids or the D-alanylation of lipoteichoic acids to polystyrene [36,37], no quantitative analysis of the impact of bacterial macromolecules on adhesion to abiotic surfaces has yet been performed at the single cell level. Therefore, it is still unclear which macromolecules of the bacterial cell wall are mainly responsible for adhesion to abiotic surfaces with highly different surface energies.

In this paper, we characterize the role of different types of surface macromolecules in the adhesion process of *S. aureus* cells to abiotic surfaces (i. e., substrates without any surface conditioning layer). Our research is based on SCFS of *S. aureus* SA113 knock-out mutant strains that exhibit changes in cell wall macromolecular properties. Their adhesion properties were investigated on hydrophilic and hydrophobic Si wafer-based substrates and compared with the corresponding data of the wild-type that was recently published [28].

Considering that the investigated groups of surface molecules are quite common in the microbial world and have several general properties (e. g., hydrophobic domains on proteins and sugar-containing backbone in teichoic acids), the outcome of this study may also be of relevance to understanding the adhesive behavior of many other bacterial species. Even for other microorganisms, such as fungi or unicellular parasites, our results may help elucidate the process of adhesion to abiotic surfaces and the role of the surface hydrophobicity of various substrates.

## 2. Results and Discussion

### 2.1. Comparing the Adhesion of Knock-Out Mutants to Hydrophobic and Hydrophilic Surfaces

To provoke highly different adhesion scenarios [28], we use two types of very smooth abiotic surfaces that we have characterized in detail: bare (hydrophilic) and silane-coated (hydrophobic) Si wafers. Adhesion studies were performed with the knock-out mutants of *Staphylococcus aureus* strain SA113. This biofilm-positive laboratory strain is a common platform to study cell wall macromolecules of *S. aureus* [31,38,39,40]. For an in-depth analysis of the cell wall macromolecular contribution to the staphylococcal adhesion process, mutants exhibiting the following changes in cell wall properties were used:

- SA113 Δ*srtA*: deficient in covalently bound cell wall proteins due to a deletion of the gene *srtA* encoding the enzyme sortase A that catalyzes the covalent linkage of proteins into the cell wall [33,41].
- SA113 Δ*tagO*: lacking the gene *tagO* encoding a glycosyltransferase that catalyzes the first committed step of wall teichoic acid (WTA) synthesis (but having lipoteichoic acids) [31].
- SA113 Δ*dltA*: lacking the gene *dltA* encoding the D-alanine-D-alanyl carrier protein ligase catalyzing the first step in the D-alanylation of lipoteichoic acids (LTAs). As a consequence, the wall and lipoteichoic acids of this mutant lack D-alanine, leading to an increased negative surface charge of the cell wall [39].

**Figure 1:**
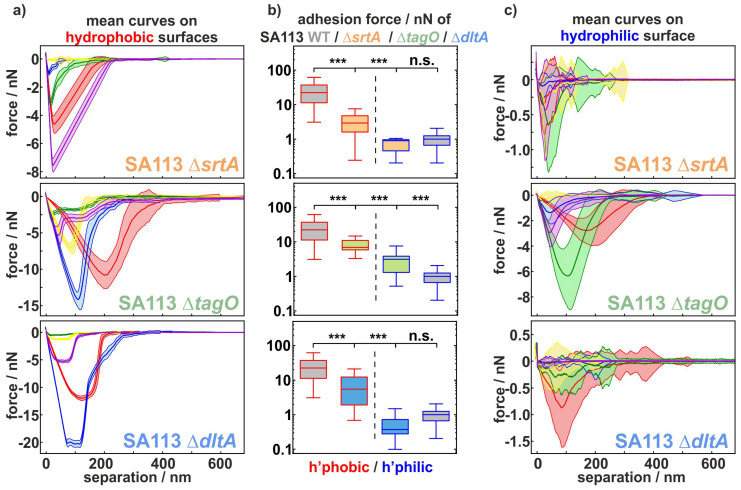
Adhesive strength of SA113 knock-out mutants to hydrophobic and hydrophilic surfaces. (**a**,**c**) For five individual mutant cells of each type, represented by five different colours, mean retraction parts of over 50 measured force–distance curves (with standard error as the shaded area) on both surfaces are shown. (**b**) In addition, box and whisker plots (min-to-max) of the adhesion force on hydrophobic and hydrophilic surfaces are shown for all tested mutant cells compared with the SA113 wild-type (WT) cells [28] (gray) in a logarithmic scale. Comparison between data sets was performed by ordinary one-way ANOVA and Dunnett’s test for multiple comparison as well as an unpaired t-test for the pairwise comparison between the adhesion of the mutants to hydrophobic and hydrophilic surfaces: n.s., *p* > 0.05; ***, *p* < 0.001.

In order to analyze differences in the adhesion of SA113 knock-out mutants to hydrophobic compared with hydrophilic surfaces, exemplary force–distance curves and box plots of the mean adhesion forces as shown in Figure 1 were calculated: for the SA113 Δ*srtA* cells, the retraction curves on hydrophobic surfaces are cup-shaped with a rather homogeneous, small standard deviation. In contrast, on the hydrophilic surface, the curves feature several minima and have rather broad standard deviation. While the curves overlap less on the hydrophobic surface and have distinctly different minima, they show a large overlap with minima at rather similar values on the hydrophilic surface. This observation are confirmed by the box plots of the mean adhesion forces in Figure 1 b): in the hydrophobic case, they differ between 0 nN and 8 nN, while they are in the range between 0 nN and 2 nN in the hydrophilic case. The distributions of the adhesion forces on the hydrophilic and hydrophobic surfaces therefore also display a high level of significance.

For SA113 Δ*tagO* cells, the situation is very different: The differences in curves shapes between hydrophobic and hydrophilic surfaces are much smaller, and the force scale differs by only a factor of two. Consequently, the differences in the distribution of the adhesion forces are quite small, though still significantly different.

This is contrasted by the adhesion of SA113 Δ*dltA* cells: Here, the curve shapes strongly differ between hydrophobic and hydrophilic surfaces and the force scale differs by more than one order of magnitude. The curves are cup-shaped on the hydrophobic surface with a very small, constant standard deviation, while the cells of the *dltA* deletion mutant display mostly irregular curve shapes with rather large standard deviations on the hydrophilic surfaces. However, the mean adhesion forces on the hydrophobic surface vary greatly between cells and are located between 1 nN and 22 nN, while all values on the hydrophilic surface are in the range of 0 nN to 3 nN. The difference between the distribution of adhesion on hydrophobic and hydrophilic surfaces is therefore also significant for this mutant strain.

Compared with the wild-type, all mutant strains show significantly lower adhesion forces on hydrophobic surfaces. In contrast, this is not the case on hydrophilic surfaces. Due to the generally low adhesion, no significant difference can be seen between the SA113 WT cells and the SA113 Δ*srtA* and SA113 Δ*dltA* cells. Only the SA113 Δ*tagO* cells show significantly higher adhesion forces on hydrophilic surfaces compared with the wild-type.

At this stage, several properties of the adhesion process can already be deduced: obviously, D-alanine residues of SA113 Δ*dltA* have a completely different influence on hydrophobic compared with hydrophilic surfaces. For cells without wall teichoic acids (SA113 Δ*tagO*), which type of substrate they adhere to is relatively unimportant. SA113 Δ*srtA* cells are somewhere between the other types. From the box and whisker plots, it can be stated that, for SA113 Δ*srtA* and SA113 Δ*dltA* cells, the number of adhesive molecules on the hydrophobic surface is much larger than on the hydrophilic surface. For SA113 Δ*tagO* cells, this difference seems quite small [28].

To analyze the adhesion behavior in more detail, several measures of the adhesion process of all mutant cells are compared with the measures of the wild-type from Reference [28]. The retraction part of each force–distance curve was evaluated in order to characterize the strength of adhesion. Hence, the maximum force between a surface and an individual cell (‘adhesion force’) was calculated. In addition, the ‘rupture length’ depicting the distance at which bacterium and surface lose contact was measured. Especially on hydrophobic surfaces, bacterial cells are also subject to a distinct attraction before reaching the surface, which is mediated by surface macromolecules (‘snap-in event’) [6]. We evaluated this mechanism with respect to the distance at which attraction started (‘snap-in separation’), as detailed in the next sections. Moreover, all bacterial strains used were analyzed for their surface charge to enable a more precise interpretation of the adhesion data.

### 2.2. Comparing the Surface Charge of Knock-Out Mutants to the Parental Strain

**Figure 2:**
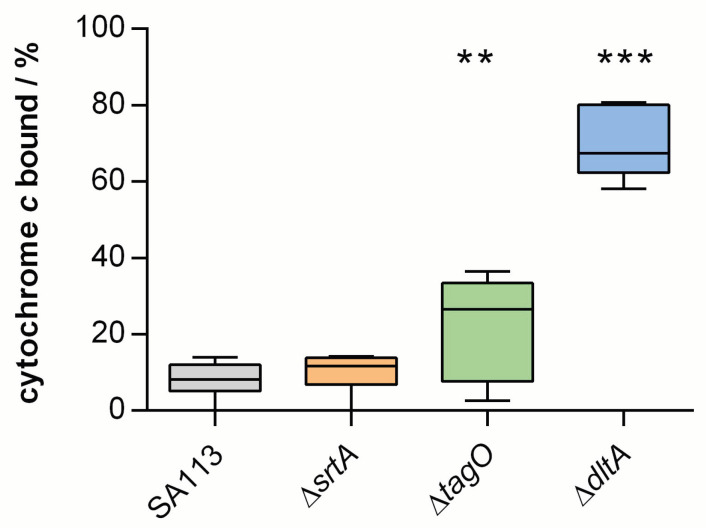
Surface charge of SA113 and its knock-out mutants. Binding capacities of the cationic protein cytochrome *c* by SA113 and its knock-out mutants. Data are presented as box and whisker plot (min-to-max) obtained from six to eight independent experiments. Comparison between data sets was conducted by ordinary one-way ANOVA and Dunnett’s test for multiple comparison: **, *p* < 0.01; ***, *p* < 0.001.

For a better understanding of how the charge of the cell surface affects bacterial adhesion, all of the bacterial strains used in this work were examined for their surface charge (see Figure 2). It is immediately apparent that all of the bacterial strains used here have a positive binding affinity to cytochrome *c* and thus possess a negative surface charge. However, there are large differences in the percentage of binding affinity: while the SA113 Δ*srtA* cells have a similar surface charge to the parental strain, the two teichoic acid mutants show an increased binding affinity to cytochrome *c*. SA113 Δ*srtA* cells lack most of the cell-wall-anchored proteins of this species, so they most likely have a lower total protein density in their cell wall, although this does not seem to change the surface charge from that of the wild-type. On the contrary, teichoic acids are considered a major factor in the formation of the overall surface charge of bacterial cells [42]. Therefore, SA113 Δ*dltA* cells lacking the positively charged D-alanine groups linked to teichoic acids carry a higher negative surface charge than wild-type cells. However, it is unknown yet whether a lack of teichoic acids directly leads to a cell surface of lower negative charge or if this lack provokes other charge-compensating effects. Notably, SA113 Δ*tagO* cells lacking the cell wall teichoic acids [31] also captured larger amounts of the cationic protein cytochrome *c* on their cell surface than SA113 wild-type (WT) cells, suggesting an increased negative surface charge, although a rather large inter-repeat variation was noticed with this mutant. This large variation in SA113 Δ*tagO* cells may also only be explained by the absence of teichoic acids, which account for up to 40% of the cell wall biomass. The lack of wall teichoic acids could leave space for additional proteins to bind to the peptidoglycan and/or adhere to the cell wall at sites usually occupied by teichoic acids, leading to greater variation in protein composition at the bacterial cell surface.

### 2.3. Statistical Analysis of Mutants Adhesion to Hydrophobic Substrates

**Figure 3:**
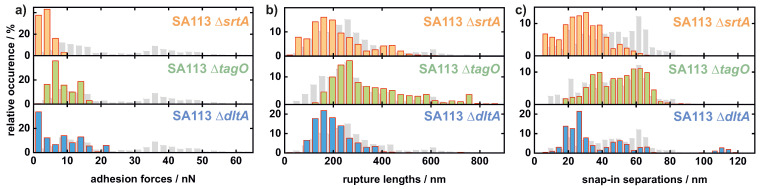
Histograms of all measured values of adhesion force (**a**), rupture length (**b**), and snap-in separation (**c**) of 18 single SA113 Δ*srtA*, 16 single SA113 Δ*tagO*, and 17 single SA113 Δ*dltA* cells on hydrophobic surfaces. Additionally, the corresponding values of the SA113 WT population are shown in gray [28].

In line with our recent observations indicating a major impact of cell wall proteins on the adhesion force of *S. aureus* (wild-type, see gray bars in Figure 3) to hydrophobic surfaces [6,28], we observed markedly reduced adhesion forces of the SA113 Δ*srtA* mutant (see Figure 3a), which lacks the majority of covalently bound proteins on the bacterial cell wall. Interestingly, the adhesion strength of SA113 cells to the hydrophobic surface is also markedly affected, when the teichoic acid composition of the bacterial cell wall is altered. Therefore, both teichoic acid mutants (SA113 Δ*tagO* and SA113 Δ*dltA*) show slightly higher adhesion forces than the SA113 Δ*srtA* mutant with values around 10 nN.

Moreover, similar to the SA113 wild type, the histograms of the rupture lengths of all mutant cells show an extended rupture length range (see Figure 3b). For the SA113 Δ*srtA* and SA113 Δ*dltA* cells, most of the values are in the range of 100–200 nm and are thus smaller than the ones seen with SA113 WT cells (200–300 nm). In contrast, the majority of values for the SA113 Δ*tagO* cells are at slightly higher values of almost 300 nm. Regarding the maximum range of the measured distribution of rupture lengths, the highest values measured for SA113 Δ*srtA* (400–500 nm) and SA113 Δ*dltA* cells (approx. 400 nm) are smaller than for the wild type (approx. 650 nm). The SA113 Δ*tagO* cells exhibit the highest values here again, with another maximum at a rupture length of 750 nm. This means that the rupture lengths of SA113 Δ*srtA* and SA113 Δ*dltA* are slightly reduced compared with SA113 WT cells while SA113 Δ*tagO* cells display on average slightly increased rupture lengths.

Of note, all mutant cells show a distinct snap-in event in the approach part of their force–distance curves so that a snap-in separation was determined (see Figure 3c). For SA113 Δ*srtA* cells, the snap-in separations are in the range of 10–60 nm and are thus considerably smaller than the ones seen with SA113 WT cells. For SA113 Δ*tagO* cells, a distribution of snap-in separation very similar to that seen for cells of the parental strain can be observed. The histogram of snap-in separations of SA113 Δ*dltA* cells features several maxima: The first at around 25 nm and the second at around 50 nm are the most prominent, but even values as high as 110 nm, which is even higher than the values seen with SA113 WT cells, can be observed. The changed shape of the histogram indicates a strong effect due to the inactivation of *dltA*.

The most striking observation on the hydrophobic surface is the markedly reduced adhesive strength of all mutants compared with the wild-type cells. This suggests that covalently bound cell wall proteins as well as wall teichoic acids and the properties of D-alanine groups in teichoic acids, have a strong—direct or indirect—influence on the strength of adhesion to this type of surface.

Considering relevant forces, we can again state that electrostatic interactions seem to play a minor—or rather indirect—role in adhesion to hydrophobic surfaces that feature a negative surface potential [43]. This observation becomes particularly evident when analyzing the surface charge (see Figure 2) in comparison with the adhesion force of SA113 WT cells and the mutants. Although SA113 Δ*srtA* cells have a surface charge comparable with the wild-type, the adhesion is strongly reduced. Thus, charge effects cannot be responsible for the reduced adhesion on hydrophobic surfaces and the answer is rather found in the absence of most cell-wall-anchored proteins. Assuming that a lower protein density in the bacterial cell wall directly leads to a lower number of tethering macromolecules during the adhesion process, the lower adhesion strength of SA113 Δ*srtA* cells fits well with our hypothesis that proteins are a major factor affecting bacterial adhesion to hydrophobic surfaces [6,28].

However, looking at the two teichoic acid mutants, a putative role of bacterial cell surface charge in adhesion to hydrophobic surfaces becomes apparent.

The higher negative surface charge of SA113 Δ*dltA* and SA113 Δ*tagO* cells might account for the strongly reduced adhesion on hydrophobic surfaces [39]. However, SA113 Δ*tagO* cells do not show a large variation in adhesion strength, as is seen for the charge (see Figure 2), which would be anticipated if its adhesion were mainly influenced by surface charge. When compared, the two teichoic acid mutants also show no major differences in adhesion forces, but there are clear differences in cytochrome *c* binding affinity. This implies that electrostatic interaction does not dominate bacterial adhesion to hydrophobic surfaces but may play a role to some extent.

**Figure A1:**
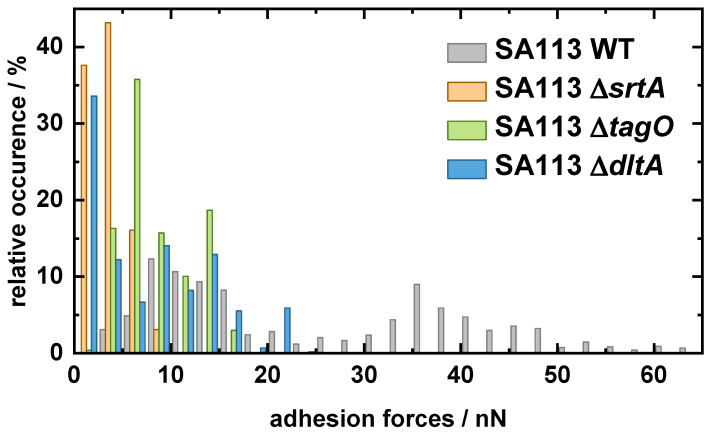
Overview histogram of all adhesion forces on OTS of 18 single SA113 Δ*srtA*, 16 single SA113 Δ*tagO*, and 17 single SA113 Δ*dltA* cells. Additionally, the corresponding values of the SA113 wild-type population are shown in gray [28].

Very interestingly, no matter what type of surface macromolecules is knocked out, the adhesion capability is reduced to a quite large extent: the SA113 Δ*srtA* cells exhibit only one seventh to one eighth of the adhesion force of the SA113 WT cells, while both teichoic acid mutants exhibit only one third of the force of the wild-type cells (see Figure A1). A likely explanation for this observation is that the absence of a class of macromolecules leave an altered environment for the remaining macromolecules (e.g., steric hindrance). Nevertheless, covalently bound surface proteins seem to have the biggest influence on adhesive strength [44,45,46].

It also cannot be excluded that the lack of cell wall teichoic acids or even of the alanine-groups of teichoic acids may change the protein composition of the cell wall and therefore alter the adhesion process. The translocation of proteins from their site of synthesis to the cell wall is a highly complex process involving many mechanisms and requires a specific interplay of charges, enzymes, and ions [47,48]. For example, cations, charge, and gradients in pH value inside the cell envelope influence protein folding, structure, and function [49]. Furthermore, the microenvironment of the cell wall is strongly influenced by the presence (or absence) of teichoic acids and, e. g., their D-alanine groups [42,50]. Moreover, it has been shown that D-alanylation directly influences protein expression [51,52,53].

After having elucidated the rather complex interplay of surface macromolecules defining adhesive strength on hydrophobic surfaces, we can now speculate about their influence on snap-in separations and rupture lengths. Most notably, the snap-in event is still observable for all mutants, whereas it it is no longer observed in SA113 WT cells in which the surface proteins have been degraded by proteases or cross-linked by glutaraldehyde [6]. Therefore, for all investigated cells, the number of surface factors can still be considered sufficient to induce this process. Additionally, the very reproducible and rather smooth shapes of the retraction curves for each individual cell (see Figure 1) supports the assumption of a rather high number of surface macromolecules participating in adhesion to hydrophobic surfaces [28].

The results of the SA113 Δ*srtA* cells show that sortase A-mediated covalently bound cell wall proteins have a stronger influence on the snap-in event, the first contact with the surface, than on the rupture length, denoting the last contact to the surface. This observation might be explained by the pure reduction in the density of surface proteins: The snap-in event occurs as soon as enough thermally fluctuating surface molecules reach the surface and bind to it. If the overall density is reduced—as it can be safely assumed for SA113 Δ*srtA* cells—this number is reached ‘later’, meaning at shorter distances to the surface, resulting in a decreased snap-in separation. The exact same reasoning holds true for the decreased rupture length: The cells lose contact at a distance where not enough proteins bind to the substrate anymore, which is probably earlier when the protein density on the bacterial cell surface is reduced than when, for fewer proteins, the force on each protein is increased, which can lead to an earlier (at smaller distance) detachment from the surface. However, the influence of covalently bound surface proteins on the rupture length is rather small. In other words, the strength of adhesion and snap-in separation on hydrophobic surfaces is largely determined by the presence of cell-wall-anchored proteins (CWAPs), but these are not necessarily the molecules that maintain final contact with the surface and thus determine the rupture length.

The slightly increased rupture lengths for SA113 Δ*tagO* cells may, without further experiments, only be explained by indirect secondary effects: For example, it is possible that at least some of the surface areas of the bacterial cell wall that are occupied by WTAs in wild-type cells are filled by more rather long CWAPs, resulting in some force–distance curves with an increased rupture length. This difference in protein composition may not have an influence on the snap-in separation because, in this case, a great number of surface proteins tether to the surface.

One bacterium out of the 17 SA113 Δ*dltA* cells shows significantly higher snap-in separation than the SA113 WT cells, with values around 110 nm. This outlier is suprising since electrostatic interactions between this mutant and the surface should generally be more repulsive than for the wild-type cells and should not lead to increased snap-in separations. However, if this outlier is to be explained, given that a SA113 Δ*dltA* mutant displays reduced activity of autolysins [54], which are known to smoothen the bacterial cell surface [55], it is conceivable that the higher roughness of the cell surface, coupled with some flexibility, could lead to more pronounced snap-in separations. Alternatively, SA113 Δ*dltA* mutants may accumulate varying amounts of extracellular milieu-localized proteins on the cell surface, such as the extracellular adherence protein Eap, which is known to bind preferentially to (poly-) anionic molecules [56].

### 2.4. Statistical Analysis of Mutants Adhesion to Hydrophilic Substrates

**Figure 4:**
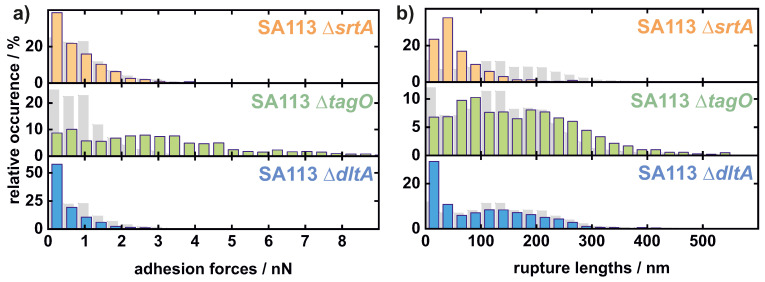
Histograms of all measured values of adhesion force (**a**) and rupture length (**b**) of 11 single SA113 Δ*srtA*, 15 single SA113 Δ*tagO*, and 18 single SA113 Δ*dltA* cells on hydrophilic surfaces. The corresponding values of an SA113 WT population are shown in gray [28].

**Figure A2:**
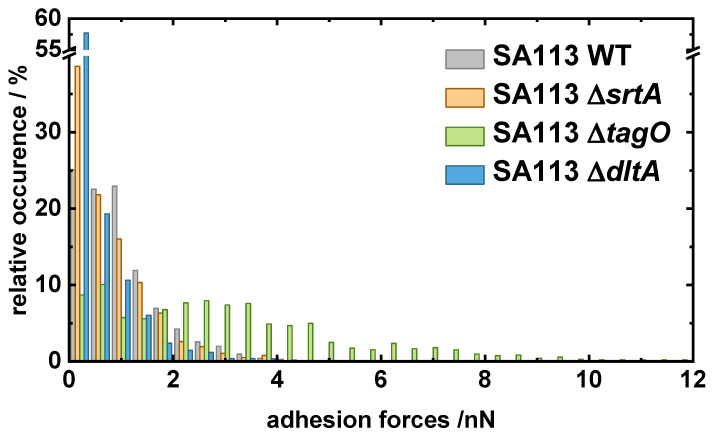
Overview histogram of all measured values of adhesion force of 11 single SA113 Δ*srtA*, 15 single SA113 Δ*tagO*, and 18 single SA113 Δ*dltA* cells on hydrophilic surfaces. The corresponding values of an SA113 wild-type population are shown in gray [28].

The principal shape of the adhesion force histograms (see Figure 4a) of SA113 Δ*srtA* and SA113 Δ*dltA* cells are similar to each other and to the histograms of the wild-type cells: Most values are located at forces near 0 nN and a smooth decay of the distribution towards higher forces can be observed. Therefore, the values of SA113 Δ*srtA* cells (about 40% of the values are close to 0 nN and the distribution ends at around 4 nN) are almost the same as the values of the SA113 WT cells (about 25%, 4 nN). SA113 Δ*dltA* cells adhere slightly less strongly to the substrate: more values (about 60%) are located close to 0 nN and the decay of the distribution is steeper than for the wild-type. In contrast, SA113 Δ*tagO* cells adhere more strongly than SA113 WT cells and the adhesion forces display a very different distribution: They have a high number of values around 3 nN and maximal values going up to 12 nN (see Figure A2).

As for the rupture lengths, the SA113 Δ*tagO* cells show slightly larger values (up to 600 nm) than the SA113 WT cells. However, the SA113 Δ*dltA* cells follow almost the same shape of distribution as the SA113 WT cells but tend to have smaller values with more than twice as many values at very low rupture lengths. The SA113 Δ*srtA* cells exhibit a distribution with a maximum below 50 nm, with occasionally higher maximal rupture lengths of about 400 nm but mainly up to 200 nm (see Figure 4b).

Adhesion forces are almost not affected by the absence of covalently bound surface proteins, whereas rupture lengths are markedly decreased. The latter implies that cell-wall-anchored proteins bind to hydrophilic surfaces and are on average longer or can be unfolded to a larger extent than other tethering macromolecules. The more minor effect of lacking covalently bound cell wall proteins on the adhesive strength to hydrophilic surfaces may be interpreted in the following way: either the covalently bound proteins have only a few hydrophilic residues (per protein or in total) able to interact with this type of surface (most likely through hydrogen bonds [28]), or there are—even without covalently bound proteins—so many surface molecules/proteins that they already occupy all possible binding sites in which the number may be limited due to interaction between different surface macromolecules. The mutant cells lacking wall teichoic acids (SA113 Δ*tagO*) show evidence for wall teichoic acids not contributing directly to the adhesion of *S. aureus* to abiotic hydrophilic substrates or at least their contribution is small compared with the adhesive strength exerted by other surface molecules. However, since we attribute bacterial adhesion solely to the binding of cell wall macromolecules [16,28,57,58], the rising adhesive strength seen with SA113 Δ*tagO* cells may also be explained by a change in protein composition due to the absence of teichoic acids. Additional proteins important for adhesion to hydrophilic surfaces on the cell surface or a higher binding affinity of the remaining cell wall macromolecules could lead to this increased adhesion force. Both hypotheses are in line with the observation that *S. aureus tagO* mutants show a higher degree of cell aggregation [36], which might be caused by unusual protein–protein interactions that are in wild-type cells precluded by WTAs. On the other hand, for SA113 Δ*dltA* cells lacking D-alanine but not the total wall and lipoteichoic acids, a reduction in adhesion is observed compared with the wild-type. This is consistent with the results made with the SA113 Δ*dltA* cells on glass [37]. This could either indicate that teichoic acids play a minor role and that this was only overshadowed by the altered cell wall macromolecule composition in the SA113 Δ*tagO* cells. However, an equally likely reason could be a charge effect. The SA113 Δ*dltA* cells are distinctly more negatively charged compared with the wild-type as well as other mutants (see Figure 2). The reason for this could be that the formation of hydrogen bonds in particular, which seem to dominate adhesion to hydrophilic substrates [28], is influenced by altered electrostatic interactions on the bacterial cell surface induced by the *dltA* mutation. The dependence of the adhesion on the charge will be investigated in future measurements.

In contrast with SA113 Δ*srtA* cells, the rupture lengths of SA113 Δ*tagO* and SA113 Δ*dltA* cells were only slightly reduced, if at all. This suggests that most likely—as described above—the on average rather long covalently bound surface proteins contribute to the adhesion of teichoic acid mutants to hydrophilic substrates and seem to make the last contact to the surface. If teichoic acids themselves bind to the surface, they usually do not contribute markedly to the rupture lengths. This might be due to the natures of both macromolecules. Teichoic acids, being composed of glycerol phosphate or ribitol phosphate, and carbohydrates linked via phosphodiester bonds most likely do not form a complex tertiary structure that is usually seen with proteins. As a consequence, teichoic acids most likely exhibit only small stretching capabilities. Proteinaceous adhesion molecules, in contrast, are usually folded to yield a complex tertiary structure important to their functionality.

In summary, for all tested knock-out mutants of *S. aureus*, we found that the adhesive strength is reduced to a large extent (about an order of magnitude) on hydrophobic surfaces, no matter which specific type this might be (see Figure 3). The adhesion of *S.aureus*—at least on hydrophobic surfaces—is thus based on a very efficient interaction of the different types of surface macromolecules investigated. This result indicates that, for theoretical modelling, the description of a protein–protein interaction of the cell wall molecules is an important task, parallel to characterizing the tethering and the detaching phases.

All experimental results obtained from mutant cells can be explained by the hypothesis that the adhesion to hydrophobic surfaces is meditated by the hydrophobic interaction between the substrate and hydrophobic residues of a large number of surface macromolecules. On hydrophilic surfaces, however, we hypothesize that a quite small number of macromolecules tether to the surface, probably by formation of directional hydrogen bonds. Hence, bond formation is slower, as can be seen by the drastically enhanced adhesive strength when applying an additional surface contact time [28]. Due to the hypothetically small number of binding macromolecules, they exhibit, in total, rather low adhesion forces. This may also explain why electrostatic interactions seem to play a role in adhesion to hydrophilic surfaces, while this effect is not present or is suppressed by the high number of tethering proteins on non-wettable surfaces due to the hydrophobic interactions.

The experimental results of SA113 Δ*srtA* cells indicate that the presence of covalently bound cell wall proteins is more important for adhesion to hydrophobic surfaces than to hydrophilic surfaces, yet they are of great relevance to the final contact (rupture length) with both surfaces. Teichoic acids and their D-alanine residues seem to influence adhesion on hydrophilic surfaces rather indirectly. The results of these mutants tend to indicate the importance of the number of binding macromolecules as well as the charge of the bacterium when adhering to hydrophilic surfaces. This effect may be caused by enhancing or reducing the probability of hydrogen bond formation. On hydrophobic surfaces, however, teichoic acids may contribute more directly through the tethering of hydrophobic D-alanine residues presented by teichoic acid regions that protrude from the cell wall.

Concerning adhesion-relevant surface proteins, we cannot state which proteins exactly contribute to which extent. Of note, different studies identified over 400 different proteins in or attached to the cell wall of *S. aureus* [59,60,61,62,63,64]. With the use of SA113 Δ*srtA* cells, we can at least state that LPXTG-anchored covalently bound proteins, although representing only a minor part of the cell wall proteome of this pathogen, have a major influence. However, other cell wall associated proteins (e. g., SERAMs, secretable expanded repertoire adhesive molecules), which might be quite high in number, likely contribute to the adhesion properties of *S. aureus* as well. Conversely, on hydrophilic surfaces, the absence of wall teichoic acids actually significantly increases adhesion, so that they probably do not play a major role in binding. This effect should be further investigated in future work. These are all crucial first steps towards understanding *S. aureus* adhesion at the macromolecule level.

## 3. Materials and Methods

### 3.1. Substrate Preparation

Si wafers (Siltronic AG, Burghausen, Germany) are the basis of the hydrophilic as well as the hydrophobic substrates used in this study. The Si substrates feature a native silicon oxide layer of 1.7(2) nm (the number in parentheses denotes the error of the last digit) and an RMS (root mean square) surface roughness of 0.09(2) nm [43]. Cleaning the Si wafers thoroughly results in a hydrophilic substrate with an advancing water contact angle of 5(2)${}^{{}^{\circ}}$, a surface energy of 64(1) mJ/m${}^{2}$, and a zeta-potential of −104.4(1) mV at pH 7.3 [43]. The hydrophobic substrate is prepared by covering a Si wafer with a self-assembled monolayer of octadecyltrichlorosilane (OTS) according to a standard protocol [65]. The result is a CH${}_{3}$-terminated substrate with an advancing (receding) water contact angle of 111(1)${}^{{}^{\circ}}$ (107(2)${}^{{}^{\circ}}$), a surface energy of 24(1) mJ/m${}^{2}$ [65], and a zeta-potential of −80.0(1) mV [43]. For force spectroscopy experiments, the substrates were immersed into phosphate-buffered saline (PBS, pH 7.3, ionic strength 0.1728 mol/L at 20 °C).

### 3.2. Bacterial Strains and Growth Conditions

All bacterial cultures used were prepared in the same way, starting the day before the force spectroscopy experiments: One colony from a blood agar plate was placed into a 5 mL tryptic soy broth (TSB) medium and incubated at 37 ${}^{{}^{\circ}}$C and 150 rpm for 16 h. On the next day, 40 μL of the overnight culture were transferred into 4 mL of fresh TSB medium and incubated for another 2.5 h to obtain exponential phase cells. Subsequently, 0.5 mL of this culture was washed three times, using 1 mL PBS each, to remove extracellular material.

### 3.3. Cytochrome c Binding Assay

In order to characterize the surface charge of SA113 and its knock-outs, cytochrome *c* binding assays [66] were carried out. Bacterial cells were cultured to the exponential growth phase as outlined above. For the cytochrome *c* binding assays, bacterial cells from exponential growth phase cultures were collected by centrifugation, washed twice with morpholinepropanesulfonic acid (MOPS) buffer (20 mM, pH 7.0), and resuspended in the same buffer to an optical density at 600 nm (OD${}_{600}$) of 7. The resulting cell suspension were incubated for 10 min at RT with 0.25 mg/mL cytochrome *c* (Merck, Darmstadt, Germany), the bacterial cells were subsequently removed by centrifugation, and the amount of cytochrome *c* that remained in the supernatant was quantitated photometrically at 530 nm using a standard curve as the reference.

### 3.4. Single-Cell Force Spectroscopy

Single bacterial probes were prepared according to a standard protocol [20]: Tipless cantilevers (MLCT-O, Bruker-Nano, Santa Barbara, USA) were covered with a thin layer of polydopamine by polymerization of dopamine hydrochloride (99%, Sigma-Aldrich, St. Louis, MI, USA) in TRIS buffer (pH 8.4). Afterwards, single bacterial cells were attached to the polydopamine-coated cantilever using a micromanipulator; care was taken so that the cells never dry out during probe preparation or force measurements. The cantilevers were calibrated before each measurement.

Force spectroscopy measurements with single bacterial probes were conducted under ambient conditions in phosphate buffered saline (PBS, pH 7.3) using a Bioscope Catalyst and a Nanowizard 4 (Bruker Nano GmbH, Berlin, Germany). Force–distance curves were performed using parameter values that correspond to similar studies [16,67,68,69]: The ramp size was 800 nm, the force trigger (denoting the maximal force with which the cell is pressed onto the substrate) was 300 pN, and retraction speed was 800 nm/s. The approach speed was 800 nm/s for force–distance measurements without surface delay and 100 nm/s when a surface delay of 5 s was applied. Surface delay times of a few seconds are a common choice to study the influence of the contact time on bacterial adhesion processes [19,58,68,69,70]. Measurements without surface delay yield a contact time below 0.5 s [16,67].

Force–distance measurements with single, viable bacterial cells were performed on either a hydrophobic or a hydrophilic substrate. Thus, for each bacterial probe and parameter set, at least 50 force–distance curves were recorded.

## 4. Conclusions

We investigated the adhesion process of *S. aureus* to abiotic substrata by combining AFM-based single cell force spectroscopy with a set of isogenic knock-out mutants. As substrates, we used a smooth silicon wafer in its natural hydrophilic oxidized state as well as covered with a self-assembling monolayer of hydrophobic silanes. On both surfaces, bacterial adhesion can be described by the binding of thermally fluctuating bacterial cell wall macromolecules.

The experiments revealed that (i) the influence of bacterial cell wall macromolecules mediating adhesion differs depending on the substrate’s hydrophobicity; (ii) on hydrophobic substrates, all tested knock-out mutants of *S. aureus* adhere about an order of magnitude less than the wild-type; (iii) covalently bound cell wall proteins largely contribute to the adhesion force on hydrophobic substrates but less on hydrophilic ones; (iv) on hydrophilic substrates, adhesion forces are influenced by the number of tethering molecules and by the cell surface charge; (v) teichoic acids seem to play a minor role for the adhesion on hydrophilic substrates; and (vi) on the hydrophobic substrates, wall teichoic acids seem to contribute directly and/or indirectly to the adhesion because of their hydrophobic residues.

In particular, the impact of electrostatic interactions through surface charges on hydrophilic substrates may be an important topic for material engineering and is of interest to future studies. With the experiments presented, it is not possible to determine which specific proteins are relevant for adhesion to abiotic surfaces and how numerous they are. This could be the subject of future studies using more sophisticated mutant cells, in which, for example, only one specific cell-wall-anchored/associated protein is knocked out, combined with qualitative simulations of the bacterial cell wall in different surface potentials.

Finally, the fundamental mechanisms of *S. aureus* adhesion to abiotic surfaces revealed in this study may be transferred to other bacterial species as microbial adhesion might generally rely on the binding of surface macromolecules. The challenge here is to design theoretical models and simulations that include the description of a protein–protein interaction of the different cell wall molecules. In addition to the characterization of the attachment and detachment phases, this is an important step towards a comprehensive biophysical understanding of biofilm formation.

## Abbreviations

AFM	Atomic force microscopy
SCFS	Single-cell force spectroscopy
WTA	Wall teichoic acid
LTA	Lipoteichoic acid

## Appendix A. Comparison of All Cells

### Appendix A.1. Comparison of All Cells Measured on Hydrophobic Surfaces

For better comparability of the *S. aureus* mutants among each other, all adhesion forces of the mutant cells measured on OTS were plotted in one histogram. In addition, they were also compared with the measurements of the wild-type from Reference [28].

### Appendix A.2. Comparison of All Cells Measured on Hydrophilic Surfaces

For better comparability of the *S. aureus* mutants among each other, all adhesion forces of the mutant cells measured on native Si were plotted in one histogram. In addition, they were also compared with the measurements of the wild-type from Reference [28].
